# Thermal Transmission Comparison of Nanofluids over Stretching Surface under the Influence of Magnetic Field

**DOI:** 10.3390/mi13081296

**Published:** 2022-08-11

**Authors:** Mubashar Arshad, Hanen Karamti, Jan Awrejcewicz, Dariusz Grzelczyk, Ahmed M. Galal

**Affiliations:** 1 Department of Mathematics, University of Gujrat, Gujrat 50700, Pakistan; 2 Department of Computer Sciences, College of Computer and Information Sciences, Princess Nourah Bint Abdulrahman University, P.O. Box 84428, Riyadh 11671, Saudi Arabia; 3 Department of Automation, Biomechanics and Mechatronics, Lodz University of Technology, 90-924 Lodz, Poland; 4 Department of Mechanical Engineering, College of Engineering in Wadi Alddawasir, Prince Sattam Bin Abdulaziz University, P.O. Box 18734, Wadi Addawasir 11942, Saudi Arabia; 5 Production Engineering and Mechanical Design Department, Faculty of Engineering, Mansoura University, Mansoura P.O. Box 35516, Egypt

**Keywords:** water, hall effect, 3D flow, hybrid nanofluid, stretching surface, magnetic field effect

## Abstract

Heat transfer at industrial levels has been revolutionized with the advancement of nanofluid and hybrid nanofluid. Keeping this development in view, this article aims to present the rate of heat transfer for conventional and hybrid nanofluids, incorporating the Hall Effect over a stretchable surface. The flow governing equations are obtained with the help of suitable assumptions, and the problem is attempted with the boundary value problem technique in MATLAB. The highly non-linear partial differential equations are transformed into non-dimensional forms using suitable similarity transforms. The criterion of convergence for solution or tolerance of a problem is adjusted to 10^−7^. Water is considered as a base fluid; copper (Cu) and silver (Ag) nanoparticles are mixed to obtain nanofluid. This novel work is incorporated for conventional and hybrid nanofluid with the effect of Hall current above the stretching/shrinking surface. Increasing the Stefan blowing parameter reduces the flow rate; it increases the heat transfer rate and nano-particle concentration of conventional and hybrid nanofluid. Both velocity components decreases by increasing the magnetic field. The Hall Effect also decreases the velocity of nanofluid. The outcomes are compared to previously published work, demonstrating that the existing study is legitimate. The heat transfer rate of the hybrid nanofluid is higher than the convential nanofluid. This study suggests more frequent use of hybrid nanofluid because of high heat transfer rates and reduced skin friction.

## 1. Introduction

When an electricity-passing fluid is oxidized with high intensity of the applied magnetic field, the normal magnetic field strength is decreased because of the free swirling of cations and anions around the magnetic lines of force. In such an incident, a current known as a Hall current is created in a normal direction to both the electric and magnetic fields. The effects of Hall currents cannot be ignored whenever the magnetic field intensity is high. In a 2D system, the Hall effect creates a cross-flow, producing the 3D flow. The effect of Hall currents on the outcomes of hydrodynamical problems is interesting and essential to understand. Hall accelerators, flight magnetohydrodynamics, refrigeration loops, and electricity transformers all use magneto-hydrodynamic flows with the Hall effect. Numerous astrophysical and geophysical conditions, as well as flows of laboratory plasmas, MHD power generation, accelerators, and other situations all, involve the effects of Hall current.

The subject of the magneto-hydrodynamic flow and heat transfer of a viscous, electrically conducting, and incompressible fluid across an unsteady semi-infinite stretched sheet is studied by Shateyi and Motsa [1]. Hayat et al. [2] examined the effects of viscous dissipation on mixed convection 3D flow of Jeffery fluid over a perpendicular stretchable surface, considering the effects of Hall and ions. Shah et al. [3] inspected the Hall effect on titanium nanofluid thin-layer flow and the thermal radiation effect with different base fluids on an inclined rotating surface. With the Hall effect, Abdelaziz [4] investigated laminar boundary layer magneto-hydrodynamic slip flow through a stretchable surface in a water-based nanofluid. Hayat et al. [5] inspected the Hall effect on the peristaltic transmission of dual stress fluid in a inclined cavity. Using Hall currents, Gaffar et al. [6] studied non-isothermal, magneto-hydrodynamic free convective boundary layer flow, heat, and mass transfer of non-Newtonian Eyring–Powell fluid from a perpendicular surface in a non-Darcy, isotropic, identical porous medium. Ahmed and Zueco [7] used hall current to model the perpendicular rotating porous channel for heat and mass transmission analysis. Awan et al. [8] explored the micropolar nanofluid between parallel plates using the Hall current effect.

Surfaces and interfaces have been historically good research topics for researchers, especially in fluid dynamics. The discovery of nanoparticles has changed the researcher’s attention dramatically. Currently, the intention of researchers is shifting to advancements in the knowledge of basic and practical features of nanoparticles and nanofluids, allowing scholars and involved engineers from many fields to connect and share their newest findings.

The notion of a nanofluid has been offered in recent years to improve the performance of heat transmission rates in currently employed fluids. First, Choi [9] presented the concept of nanofluid by suspending nanometer-sized nanoparticles to increase the thermal conductivity of fluids, such as oil and ethylene glycol, etc. Hussain et al. [10] investigated rotating nanofluid flow over a stretchable surface with a magnetic effect. Naseer et al. [11] explored the importance of the thickness of phase transition materials in thermal management. Sajjad et al. [12] gave a review on progress and prospects for personal heat management. Khan [13] investigated the Brownian motion parameter in the nano bio-convection model to check the enhancement of thermal conductivity and causes of resistance to microbe flow. Bahiraei and Heshmation [14] studied the capabilities of graphene-based nanofluids, as well as improvements in preparation procedures, stability analyses, and the types of surfactants employed. They discussed future studies, as well as thermophysical characteristics, hydrodynamic features, boiling and convective heat transport, heat exchangers, energy storage, artificial intelligence (AI), and molecular dynamics. Ejaz et al. [15] gave a review on T-junction geometry branching evolution. Arshad et al. [16] described the effects of source power and process time on pure and mixed plastic conversion. Lin et al. [17] considered the internal heat generation above a stretchable surface unsteady nanofluid flow. Akbar et al. [18] studied the radiative effect on the stagnation point flow for the stretchable surface. Khan et al. [19] used the rheologic equations of an isotropic Williamson and Casson nanofluid to investigate flow, heat transmission, nanoparticle concentration, and gyrotactic microorganisms. Bahiraei [20] gave a review of previous works on nanofluids, considering particle flow, simulation of molecular dynamics, and other theoretical investigations using the Buongiorno model. He found that particle flow is one of the reasons for discrepancies in prior studies’ results, among other things.

Waini et al. [21] studied unsteady flow and temperature transmission in a hybrid nanofluid through a shrinking/stretching sheet, as well as temporal steadiness study of the dual solutions. Bahiraei and Heshmatian [22] made a critical review on the cooling of electronic devices with the help of nanofluid. Dalkilic et al. [23] investigated the irregular temperature transmission properties of Graphite -$SiO_{2}/H_{2}O$ hybrid nanofluid flow in a plane-smooth duct with and without quad-channel perverted tape inserts of lengths ranging from 0 to 0.42 m with fixed ratio of 5. Ahmadpour et al. [24] investigated the development of the solid/liquid boundary during the solidification of liquid metal. Ahmadi et al. [25] numerically investigated the influence of the gas diffusion layer on a polymer exchange membrane. Al-Sharafi et al. [26] investigated droplet heating and stretching hydrophobic surfaces. Jing et al. [27] explained the wet foam fluid’s non-uniform heat transfer behavior in a confined fracture channel. Gulzar et al. [28] performed a tentative analysis of the rheologic behavior and steadiness characterization of hybrid nanofluid using a two-step technique. Rosca and Pop [29] used the Buongiorno model to elaborate on the unstable boundary layer flow of nanofluids. Palwasha et al. [30] investigated with variable thermophysical characteristics thin-film fluid flow. Khan et al. [31] described the inclined magnetic effect with graphene-based nanofluid. Many researchers have worked for their interests [32,33,34,35,36,37,38,39,40,41,42,43,44,45,46,47,48,49,50,51]. Ali et al. [52] used an analytical methodology for MHD liquid movement with variable viscosity and thermal conductivity. Khan et al. [53] examined effective temperature transmission, particle static motion, and Brownian motion, which defines the effects of particle size, volumetric concentration, temperature dependence, particle kind, and base fluid combination.

The discussion is based on the examination of various fluid characteristics, but there is still a gap in the literature for the comparative study of ordinary and hybrid nanofluids with the joint effect of the magnetic and electric field (known as the Hall current effect) over stretching/shrinking surfaces. The novelty of this research is to fill this gap. The Hall effect has many applications, such as monitoring flow rate, water supply treatment, oil and gas process operations, etc. So, the innovation of this article is to simulate and resolve the problem for both types of nanofluid with prominent edge accretion or ablation, as well as the Hall effect. The leading equations are transformed into ordinary differential equations via a similarity transformation. The transformed equations are tackled by MATLAB software utilizing the boundary value problem of the fourth order (bvp4c) built-in technique. The purpose of utilizing this numerical technique is to save time, be easy to tackle and provide accurate results. The outcomes of current problems, such as skin friction, Nusselt number, and mass transfer are presented with the help of graphs throughout the study.

## 2. Materials and Methods

**Figure 1:**
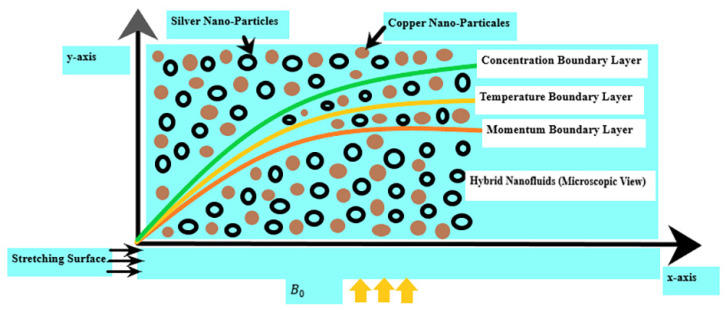
The geometry of the problem.

Consider an viscous, incompressible fluid flowing with free stream velocity$\;u_{\infty }$, temperature T, and concentration C of nanoparticles. Cu (copper) with volume fractions of $\phi_{1}=0.03$ is mixed in water to gain ordinary nanofluid and then Ag (silver) nanoparticles with volume fractions of $\phi_{2}=0.04$ are mixed to obtain a hybrid nanofluid. Perpendicular to the mass and heat transfer flow, a magnetic field $B=\left[0,\;B_{0},\;0\right]$ is applied. The intensity of the electric charge and magnetic field is considered to be at its maximum. Figure 1 depicts a schematic representation of the problem.

The problem’s basic equations in vector form for the considered fluid are given as:

(1) ∇·V=0,

(2) $$\rho \frac{\partial V}{\partial t}=div\delta +f_{b}$$

(3) $$\rho C_{p}\frac{\partial T}{\partial t}=trace\left(\delta \cdot L\right)-div\left(q\right)$$

(4) $$\frac{DC}{Dt}=D_{B}\nabla^{2}C$$

Here $\frac{DC}{Dt}$, V, and ∇ is material derivative velocity and divergence vector, respectively, ρ is density, divδ is stress tensor matrix, $f_{b}$ denoted the body force, $C_{p}$ is specific heat. T is energy change and $div\left(q\right)$ is heat flux and q=−α∇T. The following Cauchy stress tensor is used:

(5) $$\delta =-PI+\zeta ,\;\;\;\;\;\;\;\;\;\;\;\;\zeta =\mu A_{1},\;$$

(6) $$A_{1}=L+L^{t}\;$$

Here, *I* is a 3×3 identity matrix and P is the pressure, and $A_{1}\;$is Rivlin–Ericksen tensor for a first-grade fluid. The flow governing equalities are gained by solving Equations (4) and (5). The velocity vector V has components $\left(u,v,w\right)$ in their respective directions $\left(x,y,z\right)$. The Rivlin–Ericksen tensor is defined as:

(7) $$L=\left[\begin{array}{ccc} \frac{\partial u}{\partial x} & \frac{\partial u}{\partial y} & \frac{\partial u}{\partial z}\\ \frac{\partial v}{\partial x} & \frac{\partial v}{\partial y} & \frac{\partial v}{\partial z}\\ \frac{\partial w}{\partial x} & \frac{\partial w}{\partial y} & \frac{\partial w}{\partial z} \end{array}\right],\;L^{t}=\left[\begin{array}{ccc} \frac{\partial u}{\partial x} & \frac{\partial v}{\partial x} & \frac{\partial w}{\partial x}\\ \frac{\partial u}{\partial y} & \frac{\partial v}{\partial y} & \frac{\partial w}{\partial y}\\ \frac{\partial u}{\partial z} & \frac{\partial v}{\partial z} & \frac{\partial w}{\partial z} \end{array}\right],$$

(8) $$A_{1}=\left[\begin{array}{ccc} 2\frac{\partial u}{\partial x} & \frac{\partial u}{\partial y}+\frac{\partial v}{\partial x} & \frac{\partial w}{\partial x}+\frac{\partial u}{\partial z}\\ \frac{\partial v}{\partial x}+\frac{\partial u}{\partial y} & 2\frac{\partial v}{\partial y} & \frac{\partial v}{\partial z}+\frac{\partial w}{\partial y}\\ \frac{\partial u}{\partial z}+\frac{\partial w}{\partial x} & \frac{\partial v}{\partial z}+\frac{\partial w}{\partial y} & 2\frac{\partial w}{\partial z} \end{array}\right]$$

(9) $$\delta =-PI+\zeta =\left[\begin{array}{ccc} -p & 0 & 0\\ 0 & -p & 0\\ 0 & 0 & -p \end{array}\right]+\left[\begin{array}{ccc} 2\mu \frac{\partial u}{\partial x} & \mu \left(\frac{\partial u}{\partial y}+\frac{\partial v}{\partial x}\right) & \mu \left(\frac{\partial w}{\partial x}+\frac{\partial u}{\partial z}\right)\\ \mu \left(\frac{\partial v}{\partial x}+\frac{\partial u}{\partial y}\right) & 2\mu \frac{\partial v}{\partial y} & \mu \left(\frac{\partial v}{\partial z}+\frac{\partial w}{\partial y}\right)\\ \mu \left(\frac{\partial u}{\partial z}+\frac{\partial w}{\partial x}\right) & \mu \left(\frac{\partial v}{\partial z}+\frac{\partial w}{\partial y}\right) & 2\mu \frac{\partial w}{\partial z} \end{array}\right]$$

The stress components are given as:

(10) $$\begin{array}{c} \delta_{xx}=-p+2\mu \frac{\partial u}{\partial x},\;\delta_{xy}=\mu \left(\frac{\partial u}{\partial y}+\frac{\partial v}{\partial x}\right),\delta_{xz}=\mu \left(\frac{\partial w}{\partial x}+\frac{\partial u}{\;z}\right),\\ \delta_{yx}=\mu \left(\frac{\partial v}{\partial x}+\frac{\partial u}{\partial y}\right),\delta_{yy}=-p+2\mu \frac{\partial v}{\partial y},\delta_{yz}=\mu \left(\frac{\partial v}{\partial z}+\frac{\partial w}{\partial y}\right),\;\;\\ \delta_{zx}=\mu \left(\frac{\partial u}{\partial z}+\frac{\partial w}{\partial x}\right),\delta_{zy}=\mu \;\left(\frac{\partial v}{\partial z}+\frac{\partial w}{\partial y}\right),\;\delta_{zz}=-p+2\mu \frac{\partial w}{\partial z}. \end{array}$$

To ignore the induced magnetic field, it is assumed that the magnetic Reynolds number is low. The three-dimensionality of the flow is caused by the force that the Hall current effect produces in the *z*-direction, which causes a cross-movement in that direction. Furthermore, it is presumable that the study will ignore the Joule heating, viscous dissipation, and that the temperature is constant. The following are the governing equations [1] for the velocity of unsteady laminar boundary layer flows using boundary layer approximations:

(11) $$\;\frac{\partial u}{\partial t}+u\;\frac{\partial u}{\partial x}\;+\;v\;\frac{\partial u}{\partial y}=u_{\infty }\frac{\partial u_{\infty }}{\partial x}+\frac{\mu_{hnf}}{\rho_{hnf}}\frac{\partial^{2}u}{\partial y^{2}},$$

(12) $$\frac{\partial w}{\partial t}+u\;\frac{\partial w}{\partial x}\;+v\;\frac{\partial w}{\partial y}=u_{\infty }\frac{\partial u_{\infty }}{\partial x}+\frac{\mu_{hnf}}{\rho_{hnf}}\frac{\partial^{2}w}{\partial y^{2}},$$

Generalized Ohm’s law involving the hall current at constant temperature can be written as

(13) $$\underset{j}{\to }+\frac{\omega_{e}\tau_{e}}{B_{0}}\times \left(\underset{j}{\to }\times \underset{B}{\to }\right)=\sigma \left(\underset{E}{\to }+\underset{V}{\to }\times \underset{B}{\to }\right)$$

Here, $\underset{j}{\to }$ is the current density vector having components in respective directions. $\underset{E}{\to }$ is the electric field intensity vector, $\omega_{e}\tau_{e},\sigma$ are electron frequency, electrical conductivity, and electron collision, respectively. This results in $j_{y}=0$ everywhere in the flow. Thus, the x and z components after simplifying take the form:

(14) $$j_{x}=\frac{\sigma B_{0}\left(u+Hw\right)}{1+H^{2}}$$

(15) $$j_{z}=\frac{\sigma B_{0}\left(Hw-w\right)}{1+H^{2}}$$

Here, $H=\omega_{e}\tau_{e}$ is the Hall constraint. Considering these assumptions, the governing equations of continuity, momentum, energy, and concentration take the form:

(16) $$\frac{\partial u}{\partial t}+u\;\frac{\partial u}{\partial x}+v\;\frac{\partial u}{\partial y}\;=u_{\infty }\frac{\partial u_{\infty }}{\partial x}+\frac{\mu_{hnf}}{\rho_{hnf}}\frac{\partial^{2}u}{\partial y^{2}}-\frac{\sigma_{hnf}B_{o}{}^{2}\left(u+Hw\right)}{\rho_{hnf}\left(1+H^{2}\right)},$$

(17) $$\frac{\partial w}{\partial t}+u\;\frac{\partial w}{\partial x}\;+v\;\frac{\partial w}{\partial y}=u_{\infty }\frac{\partial u_{\infty }}{\partial x}+\frac{\mu_{hnf}}{\rho_{hnf}}\frac{\partial^{2}w}{\partial y^{2}}+\frac{\sigma_{hnf}B_{o}{}^{2}\left(Hu-w\right)}{\rho_{hnf}\left(1+H^{2}\right)},$$

(18) $$\frac{\partial T}{\partial t}+u\;\frac{\partial T}{\partial x}+v\;\frac{\partial T}{\partial y}=\alpha_{hnf}\frac{\partial^{2}\;T}{\partial \;y^{2}}+\frac{\mu_{hnf}}{{\left(\rho c_{p}\right)}_{hnf}}\;[\frac{\partial u}{\partial y}{]}^{2},$$

(19) $$\frac{\partial C}{\partial t}+u\;\frac{\partial C}{\partial x}+v\;\frac{\partial C}{\partial y}=D_{m}\frac{\partial^{2}C}{dy^{2}}$$

Here, u,v,w are velocity components in x,y,and z directions. The Hall parameter is denoted by H. Dynamic viscosity, density, thermal conductivity, heat capacity, and thermal diffusivity of hybrid nanofluid are denoted by $\;\mu_{hnf},\;\rho_{hnf},\;k_{hnf}$,${\left(\rho C_{p}\right)}_{hnf}$, and $\;\alpha_{hnf}$, respectively.

The boundary conditions are as follows:

(20) $$u=u_{w}=\lambda u_{\infty },\;\;v=-\frac{D_{m}}{1-C_{w}}\frac{\partial C}{\partial y},\;\;w=0,\;T=T_{w},\;\;C=C_{w}\;\;\;\;\;\;\;at\;y=0$$

(21) $$u=u_{e}=u_{\infty },\;\;\;\;w\to 0,\;\;\;\;T\to T_{\infty },\;\;\;\;C\to C_{\infty }\;\;\;\;\;\;\;\;\;\;\;\;\;\;\;\;as\;y\to \infty$$

where $u_{e}$ represents the external velocity and λ is the shrinking/stretching constraint. λ<0  denotes for shrinking of the surface, λ>0 denotes for extension of the surface, and λ=0 for the stationary surface. Temperature and volumetric concentration of hybrid nanofluids at surface and infinity are denoted by $\left(T_{w},\;C_{w}\right)$ and $(T_{\infty },\;C_{\infty }),$ respectively. Thermophysical properties and relations are presented in Table 1 and Table 2, respectively.

### 2.1. Transformation

The governing equations can be transformed by using symmetry analysis [56,57,58,59]. To avoid complexity, the problem is simplified by familiarizing the following similarity transformation [29] for $\left(f,\;g\right),\;\eta ,\;\theta ,\;$and ϕ for dimensionless velocities, space parameter, temperature, and nanoparticles concentration, respectively, as:

(22) $$\psi \left(x,y,t\right)=u_{\infty }\sqrt{v_{f}t\;cos\gamma +\left(\frac{v_{f}x}{u_{\infty }}\right)sin\gamma }\;f\left(\eta \right),\;w=u_{\infty }\sqrt{v_{f}t\;cos\gamma +\left(\frac{v_{f}x}{u_{\infty }}\right)sin\gamma }\;g\left(\eta \right)$$

(23) $$u=\frac{\partial u}{\partial y},\;v=-\frac{\partial \psi }{\partial x},\;\eta =\frac{y}{\sqrt{v_{f}t\;cos\gamma +\left(\frac{v_{f}x}{u_{\infty }}\right)sin\gamma }\;}\;,\;\theta \left(\eta \right)=\;\frac{T-T_{\infty }}{T_{w}-T_{\infty }}\;,\;$$

(24) $$\phi \left(\eta \right)=\;\frac{C-C_{\infty }}{C_{w}-C_{\infty }}$$

Here, ψ is the stream function. γ is the leading accretion or ablation parameter and $v_{f}$ is kinematic viscosity. The term must be ($v_{f}t\;cos\gamma +\left(\frac{v_{f}x}{u_{\infty }}\right)sin\gamma )$ > 0.

Equation (1) is identically fulfilled by using Equations (8)–(10). The following equations are derived using information from Table 1 and Table 2 and Equations (8)–(10):

(25) $$\frac{f^{\prime \prime \prime }}{\Phi_{1}\Phi_{2}}+\frac{1}{2}\left(cos\gamma \right)\;\eta f^{\prime \prime }+\frac{1}{2}(sin\gamma )ff^{\prime \prime }-\Phi_{5}\frac{M\left(f^{\prime }+Hg\right)}{1+H^{2}}=0,$$

(26) $$\frac{g^{\prime \prime }}{\Phi_{1}\Phi_{2}}+\frac{1}{2}\left(sin\gamma \right)\;fg^{\prime }+\frac{1}{2}\left(cos\gamma \right)\;\eta g^{\prime \prime }+\Phi_{5}\frac{M\left(Hf^{\prime }-g\right)}{1+H^{2}}=0,$$

(27) $$\frac{1}{Pr}\frac{\Phi_{4}}{\Phi_{3}}\;\theta^{\prime \prime }+\frac{1}{2}\;\left[\left(sin\gamma \right)f+\left(cos\gamma \right)\;\eta \right]\theta^{\prime }+\frac{1}{\Phi_{1}\Phi_{3}}\;Ec\;{f^{\prime \prime }}^{2}=0,$$

(28) $$\phi^{\prime \prime }+\frac{1}{2}\;Sc\;\left[\left(sin\gamma \right)f+\eta \left(cos\gamma \right)\right]\phi^{\prime }=0$$

With the transformed boundary conditions:

(29) $$f=\frac{2}{Sc}\;\frac{1}{sin\gamma }\;f_{w}\phi^{\prime },\;\;\;\;f^{\prime }=\lambda ,\;\;\;\;g=0,\;\;\;\;\theta =1,\;\;\;\;\phi =1,\;\;\;\;\;\;\;\;\;\;\;\;\;at\;\eta =0$$

(30) $$f^{\prime }=1,\;\;\;\;g=0,\;\;\;\;\theta =0,\;\;\;\;\phi =0,\;\;\;\;\;\;\;\;\;\;\;\;\;\;\;\;\;\;\;\;\;\;\;\;\;\;\;\;\;\;\;\;\;\;\;\;\;\;\;\;\;\;\;\;at\;\eta =\infty$$

Here, we define:

$$\Phi_{1}={\left[1-\left(\phi_{1}+\phi_{2}\right)\right]}^{5/2}\;,\;\Phi_{2}=\phi_{1}\frac{\rho_{s1}}{\rho_{f}}+\phi_{2}\frac{\rho_{s2}}{\rho_{f}}+\left[1-\left(\phi_{1}+\phi_{2}\right)\right]\;,\;\Phi_{3}=\phi_{1}\frac{{\left(\rho c_{p}\right)}_{s1}}{{\left(\rho c_{p}\right)}_{f}}+\phi_{2}\frac{{\left(\rho c_{p}\right)}_{s2}}{{\left(\rho c_{p}\right)}_{f}}+\left[1-\left(\phi_{1}+\phi_{2}\right)\right],\;\Phi_{4}=\frac{\left(k_{s1}+k_{s2}\right)+2k_{f}\left(1-\left(\phi_{1}+\phi_{2}\right)\right)+2\phi_{1}k_{s1}+2\phi_{2}k_{s2}}{\left(k_{s1}+k_{s2}\right)+\left(2+\left(\phi_{1}+\phi_{2}\right)\right)k_{f}-\left(\phi_{1}k_{s1\;}+\phi_{2}k_{s2}\right)}\;,\;\;\Phi_{5}=1+\frac{3[\frac{\sigma s_{1}\;\phi_{1}\;+\;\sigma_{s2}\;\phi_{2}}{\sigma_{f}}-\left(\phi_{1}+\phi_{2}\right)]}{\left(2+\frac{\sigma_{s1}+\sigma_{s2}}{\sigma_{f}}\right)-\left[\frac{\sigma_{s1}\;\phi_{1}+\sigma_{s2}\;\phi_{2}}{\sigma_{f}}\right]+\left(\phi_{1\;}+\phi_{2}\right)}\;.$$

Here, $\left(\prime \right)$ denotes the derivative w.r.t η.

$M=\frac{B_{0}{}^{2}}{\sqrt{v_{hnf}t\;cos\gamma +\left(\frac{v_{hnf}x}{u_{\infty }}\right)sin\gamma }\;}\;$ is magnetic field parameter, $Pr=\frac{\mu_{f}{\left(C_{p}\right)}_{f}}{k_{f}}$ is Prandtl number, $Ec=\frac{{\left(\mu_{\infty }\right)}^{2}}{{\left(C_{p}\right)}_{f}\left(T_{w}-T_{\infty }\right)}\;$ is the Eckert number, $Sc=\frac{\mu_{f}}{\rho_{f}D_{m}}\;$ is the Schmidt number, and $f_{w}=\frac{C_{w}-C_{\infty }}{1-C_{\infty }}\;$ is the injection/suction (or Stefan blowing) parameter, respectively. It is interesting to note that for $\phi_{1}=0.00$ and $\phi_{2}=0.00\;$ the hybrid nanofluid becomes an ordinary nanofluid. If $\phi_{1}=0.00,$ Ag/water nanofluid is gained, and if $\phi_{2}=0.00,\;$ Cu/water nanofluid is obtained.

### 2.2. Quantities of Physical Interest

The local skin resistance coefficients ($C_{fx}$,$\;C_{gz}$), Nusselt number $\;N_{ux}$, and wall mass flux $\;Q_{mx}\;$ as well as other physical variables with widespread applicability in industries, should be investigated.

(31) $$\begin{array}{c} C_{fx}=\frac{\tau_{wx}}{\rho_{hnf}u_{\infty }{}^{2}},\;\;\;C_{gz}=\frac{\tau_{wz}}{\rho_{hnf}u_{\infty }{}^{2}},\\ Nu=\frac{xq_{w}}{k_{hnf}\left(T_{w}-T_{\infty }\right)},\;\;Q_{mx}=\;\frac{xq_{m}}{D_{B}\left(C_{w}-C_{\infty }\right)}, \end{array}$$

Here:

(32) $$\begin{array}{c} \tau_{wx}=\mu_{hnf}(\frac{\partial u}{\partial y}{)}_{y=0},\;\;\tau_{wz}=\mu_{hnf}(\frac{\partial w}{\partial y}{)}_{y=0},\;\\ q_{w}=-k_{hnf}{\left(\frac{\partial T}{\partial y}\right)}_{y=0},\;\;q_{m}=-D_{B}\;{\left(\frac{\partial C}{\partial y}\right)}_{y=0}. \end{array}$$

Note, that wall resistances, heat transmission, and mass transmission on the surface are represented by ($\tau_{wx}$,$\;\tau_{wz}$),$\;q_{w}$, and $\;q_{m}$, respectively. By utilizing Equation (31) in Equation (32) we obtain:

(33) $$\begin{array}{c} Re_{x}{}^{\frac{1}{2}}C_{fx}=\frac{f^{\prime \prime }\left(0\right)}{\Phi_{1}}\frac{1}{\sqrt{sin\gamma +cos\gamma \sigma_{1}}},\;\;Re_{x}{}^{\frac{1}{2}}C_{gz}=\frac{g^{\prime }\left(0\right)}{\Phi_{1}}\frac{1}{\sqrt{sin\gamma +cos\gamma \sigma_{1}}}\;,\;\\ Re_{x}{}^{-\frac{1}{2}}Nu_{x}=-\theta^{\prime }\left(0\right)\frac{1}{\sqrt{sin\gamma +cos\gamma \sigma_{1}}},\\ Re_{x}{}^{-1/2}Q_{mx}=-\phi^{\prime }\left(0\right)\frac{1}{\sqrt{sin\gamma +cos\gamma \sigma_{1}}}. \end{array}$$

Here, $Re_{x}=\frac{\mu_{x}}{\nu_{hnf}}\;$ is the local Reynolds number with $\sigma_{1}=\frac{u_{\infty }t}{x}\;$ as the dimensionless time parameter.

### 2.3. Numerical Scheme

To tackle the problem numerically, the bvp4c algorithm is utilized in MATLAB. The high-order non-linear differential equalities are converted into a set of ordinary differential equations. Initial hypotheses for new presumed variables are taken into account. The new variables are defined as follows:

(34) $$\begin{array}{c} f=y\left(1\right),\;\;\;\;f^{\prime }=y\left(2\right),\;\;\;\;f^{\prime \prime }=y\left(3\right),\;\;\;\;\;f^{\prime \prime \prime }=y^{\prime }\left(3\right),\\ g=y\left(4\right),\;\;g^{\prime }=y\left(5\right),\;\;g^{\prime \prime }=y^{\prime }\left(5\right),\\ \theta =y\left(6\right),\;\;\theta^{\prime }=y\left(7\right),\;\;\theta^{\prime \prime }=y^{\prime }\left(7\right),\\ \phi =y\left(8\right),\;\;\phi^{\prime }=y\left(9\right),\;\;\phi^{\prime \prime }=y^{\prime }\left(9\right) \end{array}$$

The following form of ordinary differential equalities is used to solve the problem at MATLAB:

(35) $$y^{\prime }\left(3\right)=\left[-\frac{1}{2}\times \left(cos\gamma \right)\times \eta \times y\left(3\right)-\frac{1}{2}\times (sin\gamma )\times y\left(1\right)\times y\left(3\right)+\Phi_{5}\times \frac{M\times \left(y\left(2\right)+H\times g\right)}{1+H^{2}}\right]\times \left(\Phi_{1}\times \Phi_{2}\right),$$

(36) $$y^{\prime }\left(5\right)=\left[-\frac{1}{2}\times \left(sin\gamma \right)\times y\left(1\right)\times y\left(5\right)-\frac{1}{2}\times \left(cos\gamma \right)\times \eta \times y\left(5\right)-\Phi_{5}\times \frac{M\times \left(Hy\left(2\right)-g\right)}{1+H^{2}}\right]\times \left(\Phi_{1}\times \Phi_{2}\right),$$

(37) $$\;y^{\prime }\left(7\right)=\left[-\frac{1}{2}\times \left[\left(sin\gamma \right)\times y\left(1\right)+\left(cos\gamma \right)\times \eta \right]\times y\left(7\right)-\frac{1}{\Phi_{1}*\Phi_{3}}\times Ec\times y^{2}\left(3\right)\right]\times pr\times \frac{\Phi_{3}}{\Phi_{4}},$$

(38) $$y^{\prime }\left(9\right)=\left[-\frac{1}{2}\times Sc\times \left[\left(sin\gamma \right)\times y\left(1\right)+\eta \times \left(cos\gamma \right)\right]\times y\left(9\right)\right],$$

Along with the following boundary conditions:

(39) $$\left.\begin{array}{c} y\left(1\right)=\frac{2}{Sc}\;\frac{1}{\sin\gamma }\;f_{w}y\left(9\right),\;\;y\left(2\right)=\lambda ,\;\;y\left(4\right)=0,\;\;y\left(6\right)=1,\;\;y\left(8\right)=1,\;at\;\eta =0\;\;\\ \;\;\;\;y\left(2\right)=1,\;\;y\left(4\right)=0,\;\;y\left(6\right)=0,\;\;y\left(8\right)=0,\;\;\;\;\;\;\;\;\;\;\;\;\;\;\;\;\;\;\;\;at\;\eta =\infty \;\; \end{array}\right\}$$

## 3. Results and Discussion

The outcomes for conventional nanofluid and hybrid nanofluids are described in this section. The effects of different parameters on the velocity, temperature, and concentration profile are discussed. $H=0.1,\;M=1.0,\;\lambda =1.0,\;Sc=1.0,\;Ec=0.1,\;f_{w}=0.001,\;\gamma =\frac{\pi }{4},\;\mathrm{and}\;Pr=6.2$ are used to conclude the impact of parameters on flow, heat, and mass transmission through graphs and results and are compared with the literature in Table 3 for validation of the solution. Although the published work [29] only looks at ordinary nanofluids, the authors’ study looks at both ordinary and hybrid nanofluids; the results of the current problem are related to those of the research [29]. However, there is considerable agreement in calculating $f^{\prime \prime }\left(0\right)$ in terms of parameter γ. The following table shows the comparison constraint γ. We are certain that our findings for the current outcomes are consistent.

### 3.1. Velocity Profile

**Figure 2:**
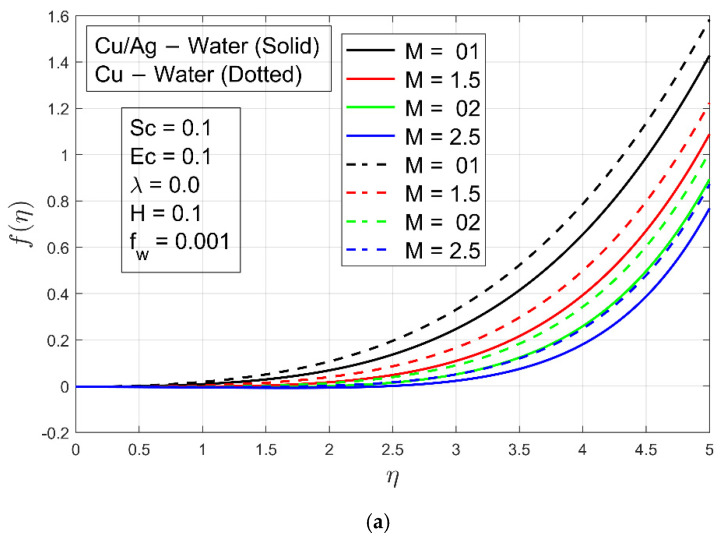

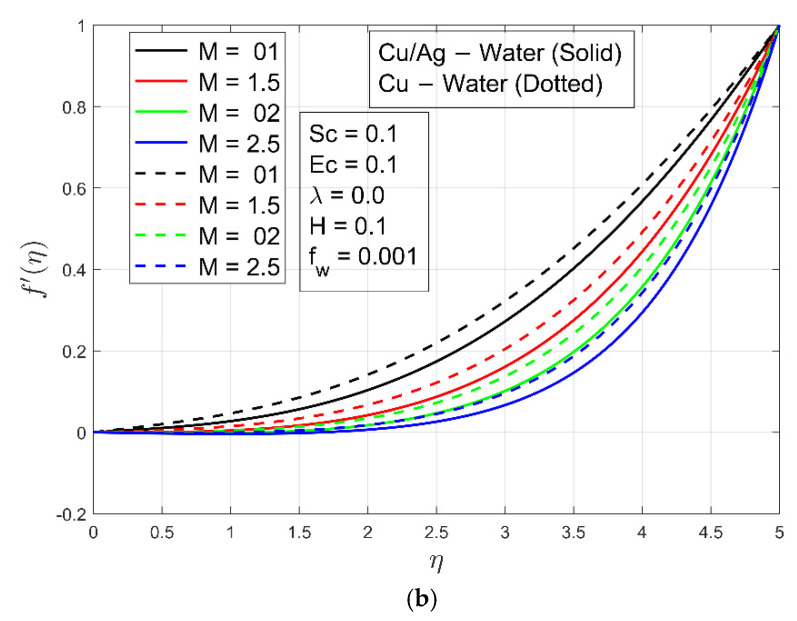
(**a**) Impact of M on velocity profile $f\;\left(\eta \right)$. (**b**) Impact of M on velocity profile $f^{\prime }\;\left(\eta \right)$.

**Figure 3:**
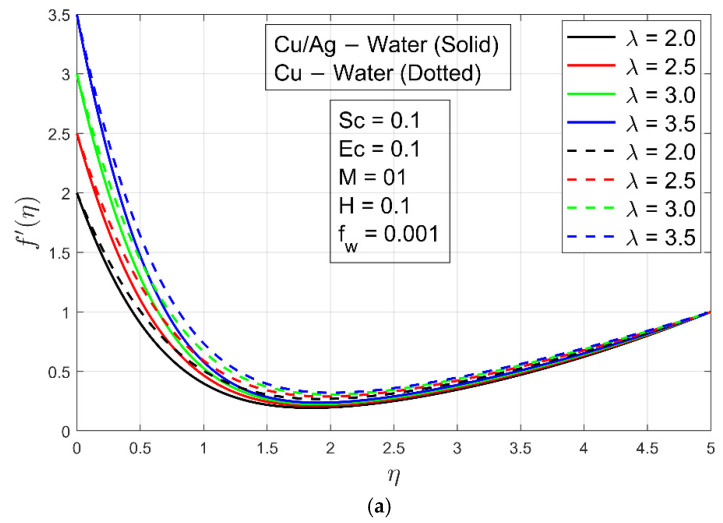

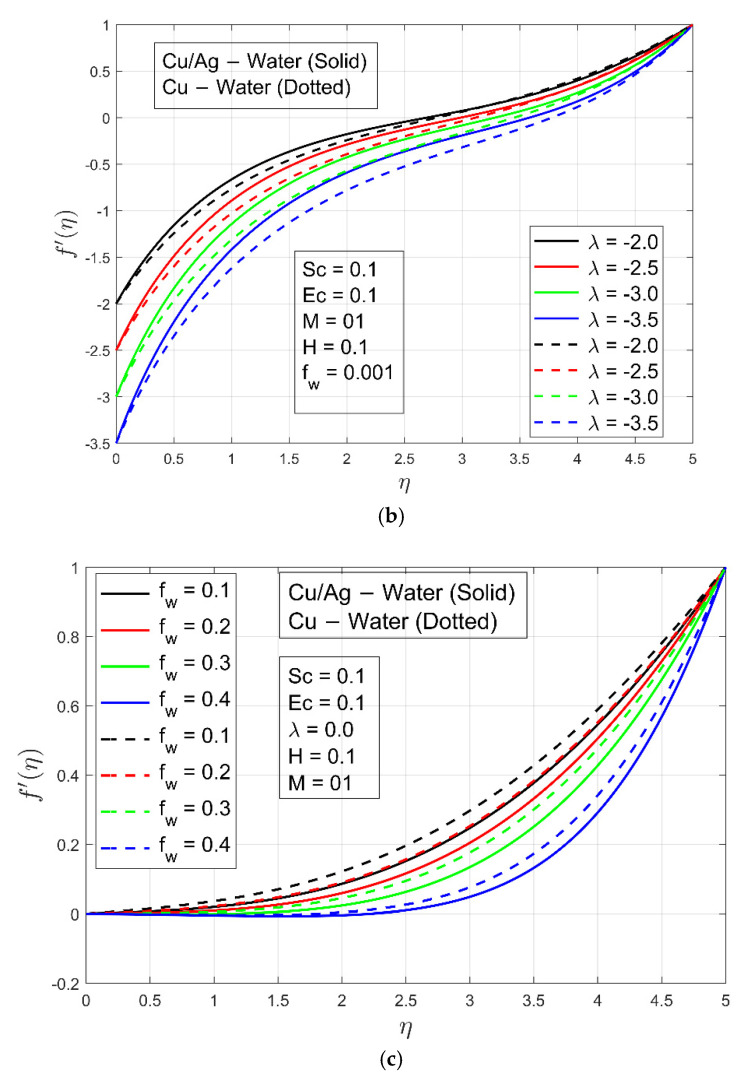
(**a**) Impact of stretching λ>0 on velocity profile $f^{\prime }\;\left(\eta \right)$. (**b**) Impact of shrinking λ<0 on velocity profile $f^{\prime }\;\left(\eta \right)$. (**c**) Impact of $f_{w}\;$ on velocity profile $f^{\prime }\;\left(\eta \right)$.

**Figure 4:**
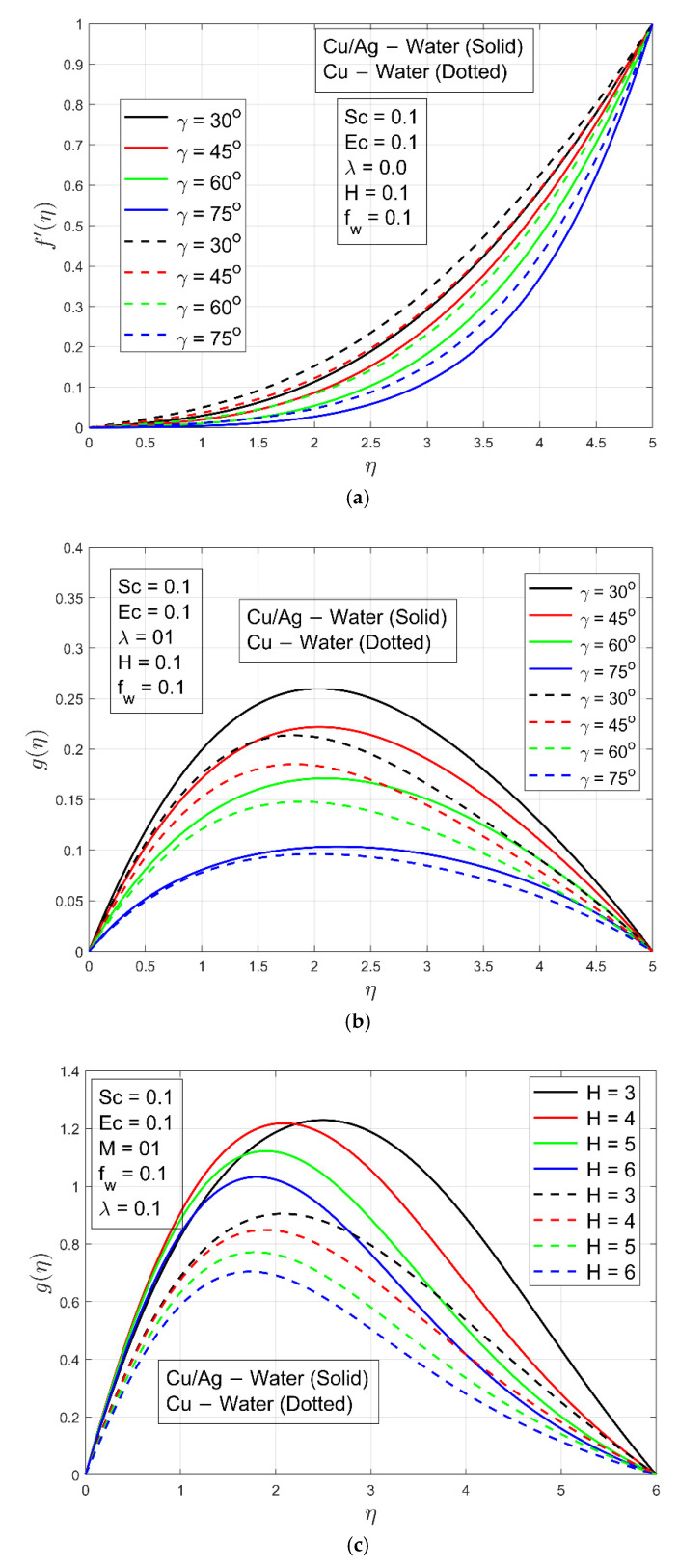

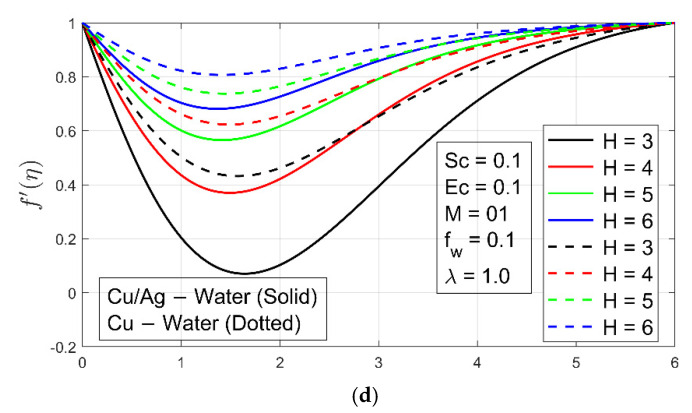
(**a**) Impact of  γ  on velocity profile $f^{\prime }\;\left(\eta \right)$. (**b**) Impact of  γ  on velocity profile $g\;\left(\eta \right)$. (**c**) Impact of  H  on velocity profile $g\left(\eta \right)$. (**d**) Impact of H  on velocity profile.

Figure 2a,b show the impacts of magnetic parameter on velocity profile $f\left(\eta \right)$ and $\;f^{\prime }\left(\eta \right),\;$respectively. These figures show that as the magnetic field parameter M is increased, the velocity of $Cu-H_{2}O$ and $Cu/Ag-H_{2}O$ decreases. The flow of both nanofluids is reduced by Lorentz forces because it resists flow. It is interesting to note that boundary layer thickness for $Cu-H_{2}O$ is smaller compared to $Cu/Ag-H_{2}O$ due to the higher density of hybrid nanofluid. In other words, $Cu-H_{2}O$ nanofluid can flow easily while $Cu/Ag-H_{2}O$ interacts with resistance. The impact of the stretching/shrinking constraint λ on velocity $\;f^{\prime }\left(\eta \right)$ is shown in Figure 3a,b. For stretching/enlarging of the surface λ>0 and λ<0 for shrinking of the surface. It is examined that flow of $Cu-H_{2}O$ nanofluid and $Cu/Ag-H_{2}O$ hybrid nanofluid is accelerated when the surface extends toward positive values of λ and decelerates when the surface shrinks (extends toward negative values of λ). On velocity, there is virtually no discernible influence. In any case, the velocity of $Cu/Ag-H_{2}O$ is lower in magnitude than$\;Cu-H_{2}O$. The reason is that $Cu-H_{2}O$ has a fast flow rate due to the presence of one nanoparticle, but $Cu/Ag-H_{2}O$ has a slower flow rate because of the presence of two nanoparticles. Furthermore, $Cu/Ag-H_{2}O$ has a higher viscosity than$\;Cu-H_{2}O$. Figure 3c depicts the influence of the suction/injection (or Stefan blowing) constraint $f_{w}$ on velocity $f^{\prime }\left(\eta \right)$. As$\;f_{w}$ increases, the velocity $f^{\prime }\left(\eta \right)$ of $Cu-H_{2}O$ and $Cu/Ag-H_{2}O$ increases. It can be perceived that the velocity $f^{\prime }\left(\eta \right)\;$ is steady in the channel’s center region. Additionally, the boundary layer thickness of $Cu/Ag-H_{2}O$ is smaller as compared to the $Cu-H_{2}O$ nanofluid because of the higher density of $Cu/Ag-H_{2}O$. Figure 4a,b displays the impact of accretion/ablation constraint γ on velocity profiles $f^{\prime }\left(\eta \right)$ and $\;g\left(\eta \right)$. As the constraint γ increases, the velocity of $Cu-H_{2}O$ and $Cu/Ag-H_{2}O$ decreases. For different values of  γ, interesting outcomes are found. It happens to accomplish a leading-edge ablation of the magnitude u cos γ by adopting the locations $\;0<\gamma <\frac{\pi }{2}$. An accretion event occurs when the condition $-\frac{\pi }{4}\leq \gamma <0$ is reached in the backward boundary layer with the rambling edge. When the cases γ=0  and $\gamma =\frac{\pi }{2}$ are implemented, the system resembles previous work, such as Blasius flat plate research and the Rayleigh–Stokes investigation in the case of hybrid nanoliquids. The Hall parameter H effect occurs to increase the magnetic field’s strength. The Lorentz force resists the flow of ordinary and hybrid nanofluid $Cu-H_{2}O$ and $Cu/Ag-H_{2}O$ and it is generated by increasing the magnetic field intensity. Figure 4c is plotted to illustrate the aforementioned fact, and it can be seen that the fluid flows are decreasing. The velocity profile $g\left(\eta \right)$ decreases, resulting in a lack of convection into the surface. The Lorentz forces exert a substantial influence on the flow, which eventually causes it to decrease. For a minimal value of the magnetic field constraint, the magnetic field is irrelevant to the flow. M is probable in that the magnetic field’s application removes the amplification of convection caused by nanoparticles right away. The non-dimensional form in the last term, i.e., $\Phi_{5}\frac{M\left(Hf^{\prime }-g\right)}{1+H^{2}}\;$ of Equation (10) proves that for low magnetic field strength, the Hall current parameter H fully controls fluid motion. So, increasing the Hall current parameter H decreases the velocity profile $g\left(\eta \right).$ Figure 4d is schemed to display the impact of Hall current constraint H  on the velocity profile $\;f^{\prime }\left(\eta \right)$. When the value of H increases, the boundary layer thickness decays because of resistance and velocity profile decays.

### 3.2. Temperature Profile

**Figure 5:**
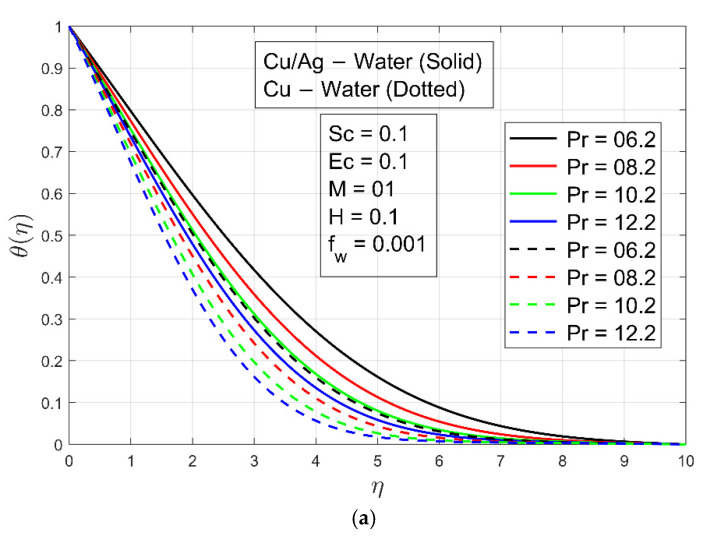

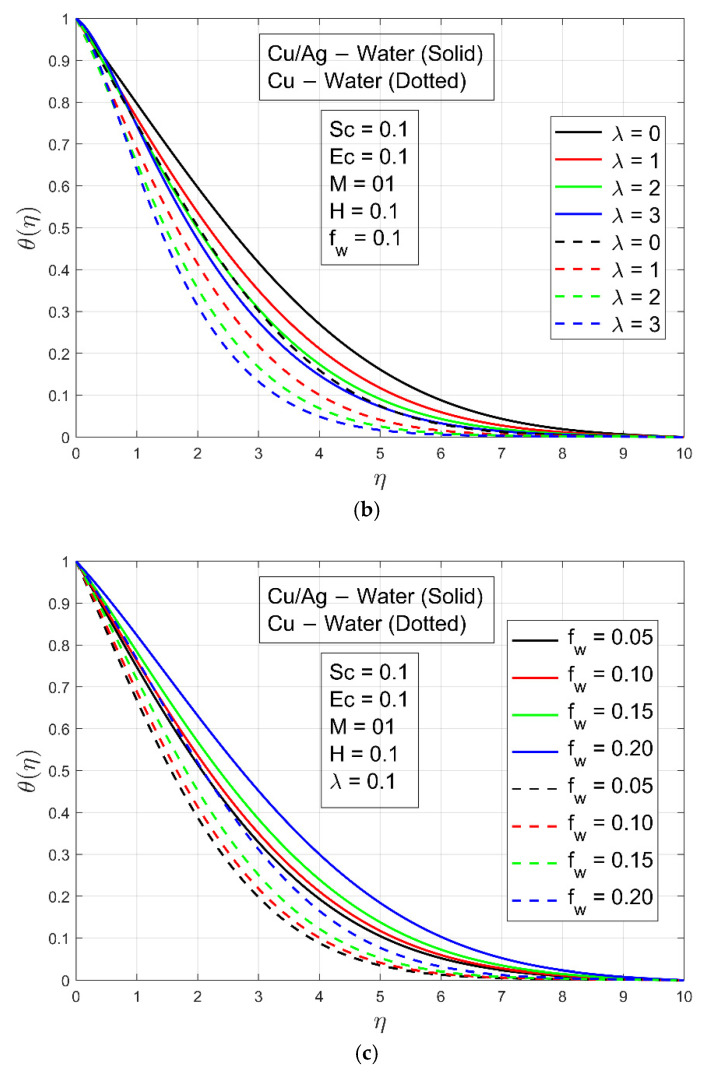
(**a**) Temperature profile $\theta \;\left(\eta \right)$ for different values of Pr. (**b**) Temperature profile $\theta \;\left(\eta \right)$ for different values of λ. (**c**) Temperature profile $\theta \;\left(\eta \right)$ for different values of $f_{w}$.

**Figure 6:**
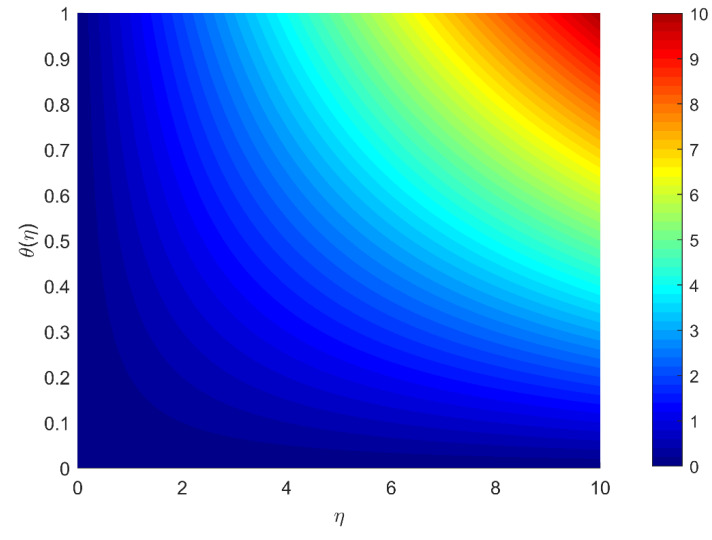
Isotherm plot under the study parameters.

Figure 5a illustrates the impact of Prandtl number Pr on the temperature profile of nanoparticles. With larger Pr values, $\;\theta \left(\eta \right)$ becomes smaller. For the whole length of the channel, viscous forces have a homogeneous effect on the heat transfer rate. It is noted that the heat transmission rate of $Cu/Ag-H_{2}O$ is higher compared to $Cu-H_{2}O$ nanofluid. Because $Cu/Ag-H_{2}O$ hybrid nanofluid has two solid nanoparticles and $Cu-H_{2}O$ has a single solid nanoparticle, the density and temperature conductivity of the hybrid nanoliquid are higher than the other nanofluid. Figure 5b represents the influence of parameter λ stretching/shrinking on temperature $\;\theta \left(\eta \right)$. For rising values of  λ, both $Cu-H_{2}O$ and $Cu/Ag-H_{2}O$ have exhibited a decrease in temperature. In stretching phenomena, the temperature of $Cu/Ag-H_{2}O$ is somewhat greater than that of $\;Cu-H_{2}O$. The temperature of $Cu-H_{2}O$ and $Cu/Ag-H_{2}O$ increases when the Stefan blowing parameter $f_{w}$ rises, as shown in Figure 5c. The reduction in heat transmission is most noticeable. Here, it is noted that the temperature transmission rate of $Cu/Ag-H_{2}O$ is higher as compared to $\;Cu-H_{2}O$ nanofluid. Figure 6 is plotted to present the isotherm for study parameters.

### 3.3. Concentration Profile, Skin Friction, Nusselt Number, and Mass Transfer

**Figure 7:**
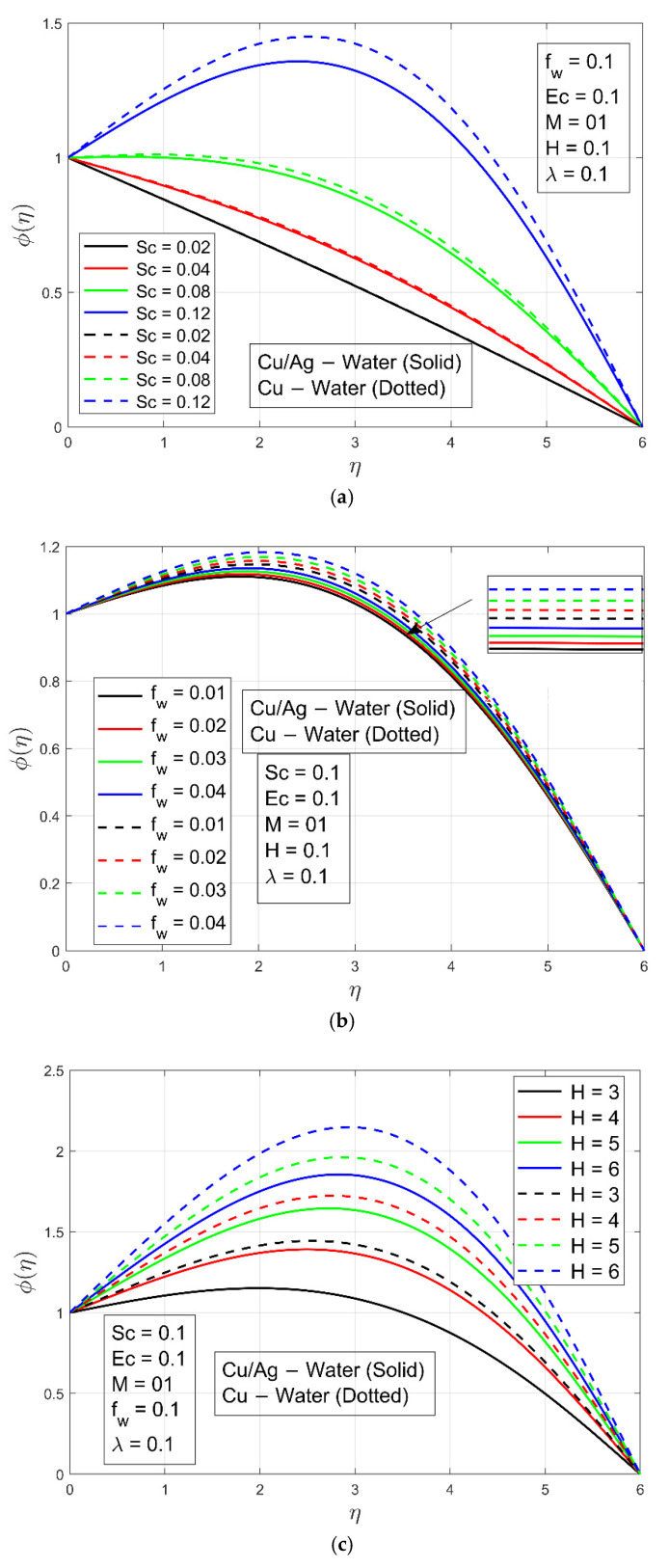
(**a**) Concentration profile $\;\phi \left(\eta \right)\;$ under the impact of Schmidt number Sc. (**b**) Concentration profile $\;\phi \left(\eta \right)\;$ under the impact of Stefan blowing parameter $f_{w}$. (**c**) Concentration profile $\;\phi \left(\eta \right)\;$ under the impact of Hall parameter H.

**Figure 8:**
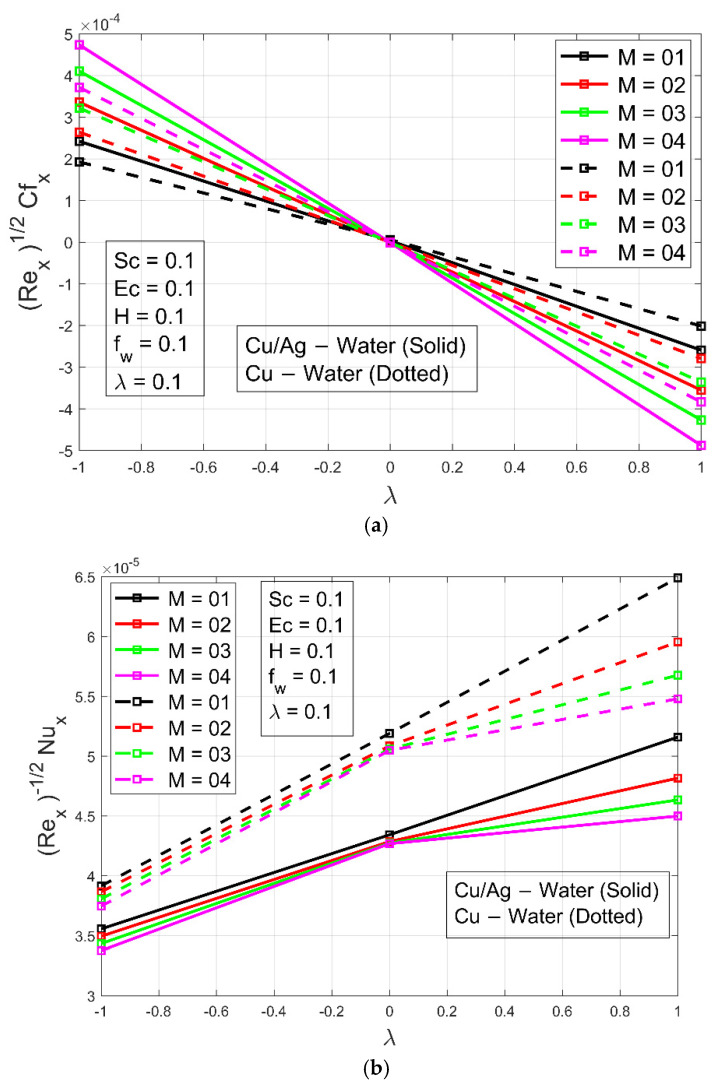

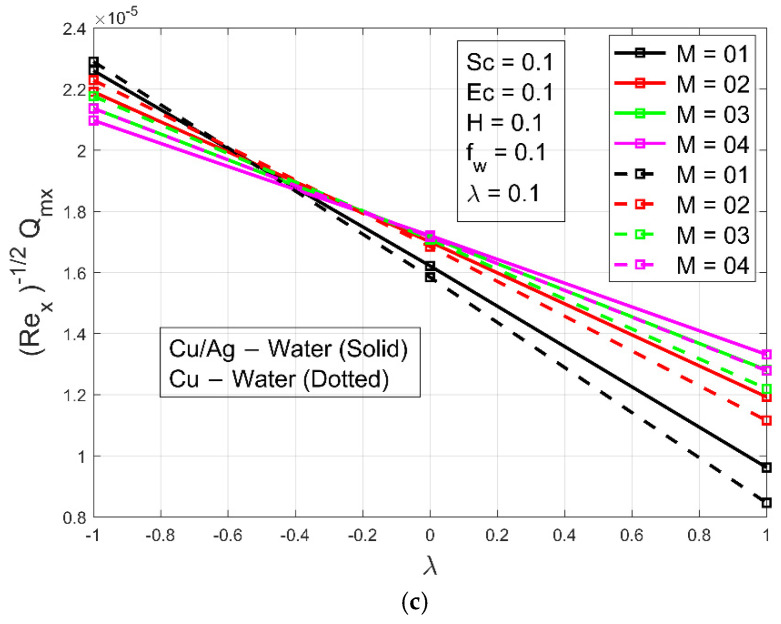
(**a**) Skin friction $Cf_{x}$ for different values of M. (**b**) Nusselt number $Nu_{x}$ for different values of M. (**c**) Mass transfer $Q_{mx}$ for different values of M.

The rise in Schmidt number Sc raises the concentration profile $\;\phi \left(\eta \right)$, as shown in Figure 7a. Viscosity increases when  Sc values are increasing. The nanoparticle concentration $\phi \left(\eta \right)\;$ is increased because viscosity is a feature of cohesive forces within various layers of $Cu-H_{2}O$ and $Cu/Ag-H_{2}O$ that have relative flow. The viscosity of the water increases as the nanoparticles are dispersed into it, resulting in a rise in the unified forces. For small values of Sc, the change is very small but as Sc  increases, the change is more visible. The influence of suction/injection constraint $f_{w}\;$ is presented in Figure 7b and has an increasing effect on the nanoparticle concentration constituting $\phi \left(\eta \right)\;$ as $f_{w}$ increases. Figure 7c illustrates the impact of the Hall constraint on concentration profile $\;\phi \left(\eta \right)$. It can be seen from Figure 7c that the concentration profile $\phi \left(\eta \right)\;$ boosts as Hall constraint H  increases. The boundary layer thickness is greater in the case of $Cu/Ag-H_{2}O$ as compared to $Cu-H_{2}O$ because of higher viscosity and of $Cu/Ag-H_{2}O$ nanofluid. Figure 8a–c shows the skin friction, Nusselt number, and mass transmission, respectively. Skin friction is higher in the case of hybrid nanofluid because of higher density as compared to nanofluid when the magnetic field constraint increases. The Nusselt number of the nanoliquid is higher as compared to the hybrid nanoliquid when the magnetic parameter increases. This is due to increasing the magnetic field parameter, which restricts the fluid from flowing. So, due to the lower density of nanofluid, the Nusselt number of the ordinary nanoliquid is higher than that of the hybrid nanoliquid. The mass transmission rate is higher for the hybrid nanofluid associated with the nanofluid as the magnetic field constraint increases.

## 4. Conclusions

This novel work is incorporated for the nanofluid and hybrid nanofluid with the Hall current effect over a stretchable surface to investigate the thermal transmission and mass transfer rates. Water is considered the base fluid while copper and silver are used to prepare nanofluid and hybrid nanofluid. The outcomes are obtained by using the boundary value problem technique at MATLAB and presented through graphs throughout the study. The major outcomes of the articles are:

1. The velocity profile reduces when the magnetic M, Stefan blowing $f_{w}$, leading-edge accretion or ablation γ, and Hall parameter H are increased.
2. The velocity profile reduces when the magnetic, Stefan blowing, leading-edge accretion or ablation parameters and Hall are increased.
3. The temperature profile decays when Prandtl number Pr and surface stretching rate λ increases but the behavior is opposite when Stefan blowing parameter $f_{w}$ increases.
4. An increase in surface stretching rate λ, increases the velocity profile of both nanofluids.
5. The concentration profile ϕ of nanofluids rises when the Hall parameter H, Stefan blowing parameter $f_{w},$ and the Schmidt parameter Sc are increased.
6. The mass transfer rate and skin friction are higher for hybrid nanofluid when magnetic parameter M increases but the Nusselt number is higher for ordinary nanofluid.
7. The heat transmission rate of the hybrid nanoliquid $Cu/Ag-H_{2}O$ is always higher than $Cu-H_{2}O$ nanofluid.

## Figures and Tables

**Table 1:** Thermo-physical characteristics of base fluid and nanoparticles [10,54].

Properties	Water (H_2_O)	Copper (Cu)	Silver (Ag)
ρ (Density)	$\rho_{f}=997$	$\rho_{s1}=8933$	$\rho_{s2}=10,500$
$c_{p}$ (Heat Capacity)	${\left(c_{p}\right)}_{f}=4179$	${\left(c_{p}\right)}_{s1}=385$	${\left(c_{p}\right)}_{s2}=235$
k (Thermal Conductivity)	$k_{f}=0.613$	${\left(k\right)}_{s1}=400$	${\left(k\right)}_{s2}=429$
σ (Electrical Conductivity)	$\sigma_{f}=5\times 10^{-2}$	${\left(\sigma \right)}_{s1}=5.9\times 10^{7}$	${\left(\sigma \right)}_{s2}=6.3\times 10^{7}$
Pr (Prandtl Number)	Pr=6.2	−	−

**Table 2:** Thermo-physical properties of conventional and hybrid nanofluid [10,55].

Properties	Nanofluid (Cu − Water)
Density $\left(\rho \right)$	$\rho_{nf}=\rho_{f}\left(1-\phi \right)+\phi \rho_{s}$
$\mathrm{Dynamic}\;\mathrm{Viscosity}\;\left(\mu \right)$	$\mu_{nf}=\frac{\mu_{f}}{{\left(1-\phi \right)}^{\frac{5}{2}}}$
$\mathrm{Heat}\;\mathrm{Capacity}\;\left(\rho c_{p}\right)$	${\left(\rho C_{\;p}\right)}_{\;nf}={\left(\rho C_{\;p}\right)}_{f}\;\left(1-\varphi \right)+\phi {\left(\rho C_{\;p}\right)}_{s}$
$\mathrm{Thermal}\;\mathrm{Conductivity}\;\left(k\right)$	$\frac{k_{nf}}{k_{f}}=\frac{k_{s}+2k_{f}-2\phi \left(k_{f}-k_{s}\right)}{k_{s}+2k_{f}+\phi \left(k_{f}-k_{s}\right)}$
$\mathrm{Electrical}\;\mathrm{Conductivity}\;\left(\sigma \right)$	$\frac{\sigma_{nf}}{\sigma_{f}}=1+\frac{3\left(\sigma -1\right)\phi }{\left(\sigma +2\right)-\left(\sigma -1\right)\phi },\;$where $\sigma =\frac{\sigma_{s}}{\sigma_{f}}$
Properties	$\mathrm{Hybrid}\;\mathrm{Nanofluid}\;\left(\mathrm{Cu}/\mathrm{Ag}-\mathrm{water}\right)$
$\mathrm{Density}\;\left(\rho_{hnf}\right)$	$\rho_{hnf}=\left(1-(\phi_{1}+\phi_{2}\right))\rho_{f}+\phi_{1}\rho_{s1}+\phi_{2}\rho_{s2}$
$\mathrm{Dynamic}\;\mathrm{Viscosity}\;\left(\mu_{hnf}\right)$	$\mu_{hnf}=\frac{\mu_{f}}{{\left[1-\left(\phi_{1}+\phi_{2}\right)\right]}^{5/2}}$
$\mathrm{Heat}\;\mathrm{Capacity}\;{\left(\rho C_{p}\right)}_{hnf}$	${\left(\rho C_{p}\right)}_{hnf}=\left[1-\left(\phi_{1}+\phi_{2}\right)\right]{\left(\rho c_{p}\right)}_{f}+\phi_{1}{\left(\rho c_{p}\right)}_{s1}+\phi_{2}{\left(\rho c_{p}\right)}_{s2}$
Thermal Conductivity $\left(k_{hnf}\right)$	$\frac{k_{hnf}}{k_{f}}=\frac{\left(k_{s1}+k_{s2}\right)+2k_{f}\left(1-\left(\phi_{1}+\phi_{2}\right)\right)+2\phi_{1}k_{s1}+2\phi_{2}k_{s2}}{\left(k_{s1}+k_{s2}\right)+\left(2+\left(\phi_{1}+\phi_{2}\right)\right)k_{f}-\left(\phi_{1}k_{s1}+\phi_{2}k_{s2}\right)}$
Electrical Conductivity $\left(\sigma_{hnf}\right)$	$\frac{\sigma_{\mathrm{hnf}}}{\sigma_{f}}=1+\frac{3[\frac{\sigma s_{1}\;\phi_{1}+\sigma_{s2}\;\phi_{2}}{\sigma_{f}}-\left(\phi_{1}+\phi_{2}\right)]}{\left(2+\frac{\sigma_{s1}+\sigma_{s2}}{\sigma_{f}}\right)-\left[\frac{\sigma_{s1}\;\phi_{1}+\sigma_{s2}\;\phi_{2}}{\sigma_{f}}\right]+\left(\phi_{1}+\phi_{2}\right)}$

**Table 3:** Comparison of present results with literature published.

*γ*	Todd [60]	Roşca and Pop [29]	Present Results	Error
15°	0.5807	0.58072	0.58073	0.00001
30°	0.5770	0.57700	0.577028	0.000028
45°	0.55287	0.55287	0.552894	0.000024
60°	0.50720	0.50721	0.507228	0.000018

## Data Availability

Not applicable.

## Nomenclature

H	Hall Effect parameter
$C_{p}$	Specific heat at constant pressure
M	Magnetic parameter
Ec	Eckert number
T,C	Temperature, concentration
Re	Reynold number
λ	Surface parameter
$C_{fx},C_{gz}$	Skin friction cofficients
k	Thermal conductivity
Nu, Pr	Nusselt and Prandtl numbers
Sc	Schmidt number
u,v,w	Velocity components in x,y,z direction
$f_{w}$	Stefan blowing parameter
f,g	Dimensionless velocity components
Greek Symbols	
α	Thermal diffusivity
μ	Dynamic viscosity
η	Similarity variable
ψ	Stream function
ρ	Density
ϕ	Nanoparticle volume fraction
$\nu_{f}$	Kinematic viscosity
θ	Temperature
γ	Accertion or ablation parameter
σ	Electrical conductivity
Subscripts	
f,nf	Fluid, Nanofluid
s	Solid nanoparticles
hnf	Hybrid nanofluid
w, ∞	Wall and free stream
